# Trapping Iron Oxide into Hollow Gold Nanoparticles

**DOI:** 10.1007/s11671-010-9792-x

**Published:** 2010-09-28

**Authors:** Chienwen Huang, Jiechao Jiang, Chivarat Muangphat, Xiankai Sun, Yaowu Hao

**Affiliations:** 1 Department of Materials Science and Engineering, University of Texas at Arlington, Arlington, TX 76019, USA; 2 Department of Radiology and Advanced Imaging Research Center, University of Texas Southwestern Medical Center at Dallas, Dallas, TX 75390, USA

**Keywords:** Gold nanoparticles, Iron oxide nanoparticles, Core/shell nanoparticles, Hollow nanoparticles, Porous nanoparticles, Plasmonics

## Abstract

Synthesis of the core/shell-structured Fe_3_O_4_/Au nanoparticles by trapping Fe_3_O_4_ inside hollow Au nanoparticles is described. The produced composite nanoparticles are strongly magnetic with their surface plasmon resonance peaks in the near infrared region (wavelength from 700 to 800 nm), combining desirable magnetic and plasmonic properties into one nanoparticle. They are particularly suitable for in vivo diagnostic and therapeutic applications. The intact Au surface provides convenient anchorage sites for attachment of targeting molecules, and the particles can be activated by both near infrared lights and magnetic fields. As more and more hollow nanoparticles become available, this synthetic method would find general applications in the fabrication of core–shell multifunctional nanostructures.

## Introduction

Gold nanoparticles (AuNPs) and superparamagnetic iron oxide nanoparticles (SPIONs) have been subjects of intensive research in the last decade [1,2]. They are generally considered as biocompatible and are of great interest for diagnostic imaging and therapeutic applications. SPIONs are currently being used as magnetic resonance imaging contrast agents in clinic. Heating effect of SPIONs in an alternating magnetic field has been extensively explored for potential hyperthermia treatment of cancer [3]. Biomedical applications of AuNPs originate from their surface plasmon resonance (SPR) effect, a strong enhancement of absorption and scattering of light in resonant with the SPR frequency, which has been utilized for photothermal ablation treatment and optical imaging. To tune the SPR wavelength to the near infrared (NIR) region that is commonly regarded as a 'clear window' for deep tissue penetration of light, various types of Au nanoparticles such as nanorods [4], nanoprisms [5], nanoshells [6,7], and nanocages [8,9] have been developed and investigated.

In recent years, combining SPIONs with Au to form a composite multifunctional nanoparticles has attracted considerable attention [10-14]. To date, the effort has been mostly limited on coating iron oxide particles with a thin layer of Au, where the Au shell not only provides convenient anchorage sites for functionalization of biomolecules through the well-established Au-thiol conjugation procedure but also protects SPIONs from dissolution and aggregation. However, by such an approach, it is difficult, if not impossible, to tune the SPR wavelengths to the NIR region. Reported core/shell particles usually have their SPR in the visible light range (from 500 to 600 nm), which limits their optical functions for in vivo applications. Here, we report an approach to construct iron-oxide/Au core/shell nanoparticles by trapping iron oxide nanoparticles into hollow AuNPs. Such nanoparticles are magnetic with their SPR peaks in the NIR region (wavelength from 700 to 800 nm).

## Experimental Methods

### Synthesis of Porous Hollow Au Nanoparticles

Synthesis of porous hollow Au nanoparticles (PHAuNPs) was detailed in the reference [15]. The key processes are listed here. The synthesis process was conducted inside a typical three-electrode electrodeposition cell with Ag/AgCl electrode in 3 M NaCl solution as the reference [U_ep_ = 0.250 V vs. standard hydrogen electrode (SHE)] and a platinum mesh as the counter electrode. A stack of two anodic alumina filtration membranes (from Whatman Corp.) with the pore diameter of about 300 nm and thickness of 60 μm were used as templates. A 700-nm-thick film of Cu was sputter-deposited to block the pores of the bottom membrane, and this acted as the working electrode for electrodeposition. A commercial Au sulfite electrodeposition solution (Techni-Gold 25 ES from Technic Inc.) was used as the electrolyte. The pH of the solution was about 7.0, which was changed to 6.0 by adding 0.4 M Ni sulfamate solution. A potential of 0.80 V (vs. Ag/AgCl reference) was applied to the working electrode using a Princeton Applied Research 273A Potentiostat/Galvanostat, at which hydrogen evolution occurred. As hydrogen molecules diffused into the pores inside the membranes, hydrogen nanobubbles formed on the inner wall surface of the pores. These hydrogen nanobubbles served as templates. The high concentration of hydrogen molecules in the bubble boundary reduced the Au^+^ complex ion to form Au clusters. Then, these clusters act as a catalyst to trigger the autocatalytical disproportionation reaction:

$$3{\text{Au}}^{1+}(\text{in}\;\text{sulfite}\;\text{complex})\overset{\text{on}\;\text{Au}\;\text{surface}}{\to }{\text{Au}}^{3+}+2\text{Au}$$

As a result, an Au shell forms around the hydrogen bubble. Metal Au evolves from clusters, particles to porous networks, forming PHAuNPs, which adhere to the inner wall surface of the pores inside both (bottom and top) membranes. The PHAuNPs-loaded top membrane was ready for the next step of loading Fe_3_O_4_ nanoparticles into these PHAuNPs after being washed by passing deionized water through the membrane several times.

### Loading Fe_3_O_4_ Nanoparticles into PHAuNPs

The formation of Fe_3_O_4_ nanoparticles via alkaline precipitation was conducted by following a previously reported procedure [16]. Briefly, anhydrous 5.2 g FeCl_3_ (0.032 mol) and 2 g FeCl_2_ (0.016 mol) were mixed in 25 ml of DI water containing 0.85 ml of 12.1 N HCl under vigorous stirring. This aqueous solution flew through the PHAuNP-loaded AAO membrane using a vacuum filtration setup, which guarantees all PHAuNPs were wetted with the solution. The membrane was removed from filtration setup and immersed into the solution for additional 30 min. The wet membrane was then transferred into 5 ml of 0.5% NH_4_OH and was allowed to sit for 20 min. The color change to yellow–orange indicates the precipitation of iron oxide particles. After the precipitation, the iron oxide nanoparticles (~10 nm) formed within the membrane were washed away by flowing DI water through the membrane using the vacuum filtration setup. The Fe_3_O_4_/PHAuNPs core/shell nanoparticles were released into water after the dissolution of membrane using 2 M NaOH solution. The particles were cleaned by several cycles of dispersion in DI water followed by centrifugation.

### Characterization of Fe_3_O_4_/PHAuNPs Core/Shell Nanoparticles

Samples for TEM were made by simply dipping a copper grid into the diluted nanoparticle water suspension. TEM micrographs were taken using a Hitachi H9500 HR-TEM. Absorption spectra of the particle water suspensions were measured using a Perkin-Elmer Lambda 19 UV/VIS/NIR Spectrometer. Hysteresis loop of dried particle powder was measured using a vibrating sample magnetometer.

## Results and Discussions

**Figure 1:**
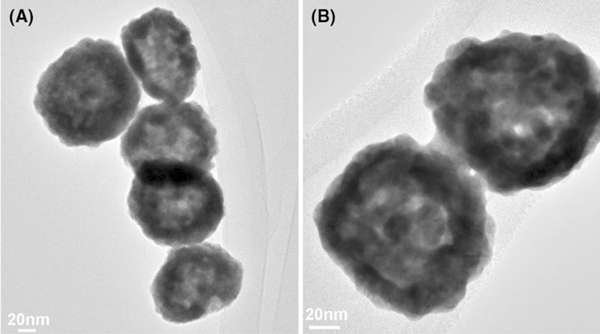
**a and b HRTEM micrographs of PHAuNPs, showing the hollow core and the porous shell with pore size about 2–3 nm**.

Synthesized PHAuNPs feature a sub-25-nm shell with a 50-nm hollow core. The shell is of porous nature with the pore size about 2–3 nm, as measured in the high-resolution transmission electron microscopy (HRTEM) image shown in Figure 1a. These nanoscale pores in the shell allow ions (Fe^2+^ and Fe^3+^) to diffuse into the hollow space in the core, where precipitation of Fe_3_O_4_ takes place upon the addition of OH^-^. The sizes of precipitated Fe_3_O_4_ nanoparticles (5–20 nm) are larger than the pore size, resulting in the trapping of the iron oxide nanoparticles inside the PHAuNPs (Figure 1b).

**Figure 2:**
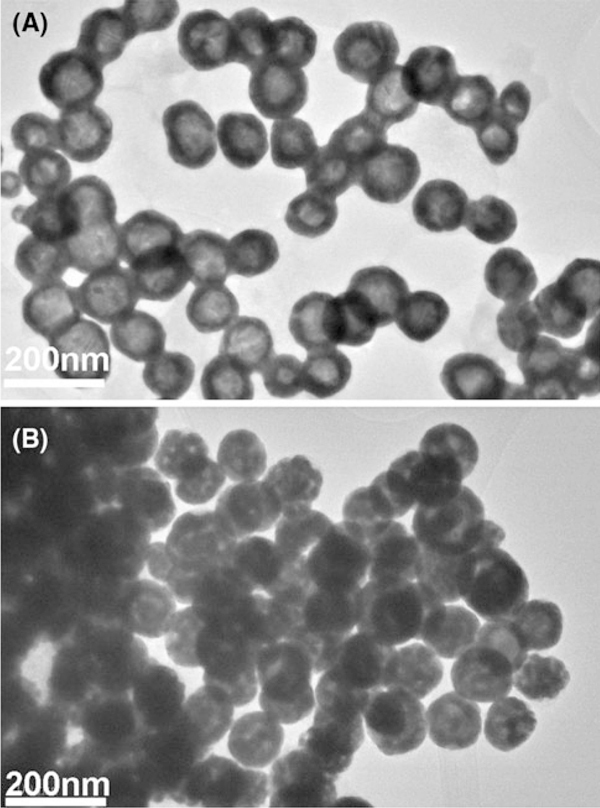
**TEM micrographs of PHAuNPs before a and after b loading iron oxide nanoparticles**.

Figure 2 shows TEM analysis before and after loading of iron oxide nanoparticles. After loading, the hollow core of PHAuNPs is occupied by solid substances. During the precipitation, Fe_3_O_4_ nanoparticles also formed outside of PHAuNPs, but TEM micrographs clearly show that no small iron oxide nanoparticles were attached to the PHAuNP surface. This is in agreement with the common notion that iron oxide usually does not stick to the Au surface [11]. Given the very different sizes of PHAuNPs (~100 nm) and non-trapped Fe_3_O_4_ nanoparticles (<20 nm), they can be readily separated using centrifugation.

**Figure 3:**
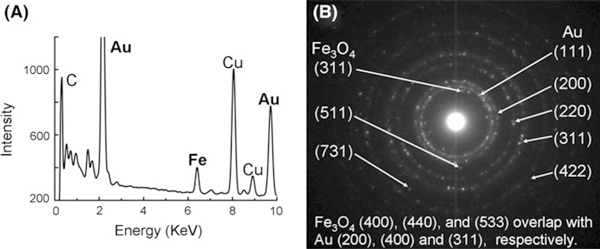
**a EDS spectrum of one single particle, showing the coexistence of Au and Fe**. **b** SAED pattern from three particles, showing a superposition of Au and Fe_3_O_4_ lattices.

The loading of Fe_3_O_4_ to the core of PHAuNPs was confirmed by energy-dispersive X-ray (EDS) analysis of one single particle and the selected area electron diffraction (SAED) pattern from three particles. EDS shows the coexistence of Au and Fe in a single particle (Figure 3a, Cu peak is from the TEM grid). The low intensity of Fe may be due to the shield effect of the thick Au shell. The SAED pattern is a superposition of Au and Fe_3_O_4_ lattices (Figure 3b), showing three distinguishable planes of (311), (511), and (731) from Fe_3_O_4_. Other Fe_3_O_4_ planes overlap with Au planes.

**Figure 4:**
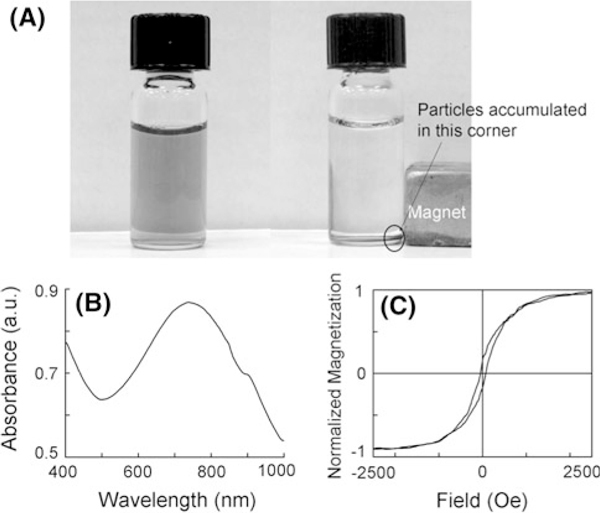
**The plasmonic and magnetic properties of the Fe_3_O_4_-loaded PHAuNPs**. **a** Appearance of a bottle of particle water suspension. The particles can be dragged toward a permanent magnet. **b** Absorption spectrum of the particle water suspension, showing a broad peak centering at 750 nm. **c** Hysteresis loop of dried particle powder, showing that the suspension consists of a mixture of superparamagnetic and ferromagnetic nanoparticles.

Shown in Figure 4a is the appearance of a bottle of particle water suspension. The cyan color indicates that the suspension absorbs red light. The absorption spectrum is shown in Figure 4b, which has a broad peak centering at 750 nm. This absorption peak corresponds to the SPR wavelength. Compared to PHAuNPs before loading iron oxide, the absorption spectrum shows little change. For core/shell nanoparticles, it is well known that the SPR wavelength is dependent on the refractive indices of medium, shell and core. Changing core material usually causes a shift of the SPR wavelength. However, PHAuNPs have a relatively thick shell (>20 nm). Through a three-dimensional finite difference time domain (FDTD) simulation (using a commercial software from Lumerical Inc), we have proved that at this thickness, the red-shifts of SPR peaks are mainly caused by their surface roughness, and the hollow nature of these particles plays only a minor role [17]. The simulation results show that SPR peaks for hollow particles are only slightly red-shifted compared to solid particles with the same outer diameter (100 nm). For particles with a roughness of 5 nm, SPR peak shifts to longer wavelength (~630 nm). As the roughness increases to 8 nm which is the average grain size in the shell, a much greater red-shift (to 720 nm) is observed. This roughness effect is due to the strong interaction of electric fields from adjacent bumps on the surface, similar to the plasmonic properties of the aggregates of several nanoparticles. The simulated results are in good agreement with experimental results. This unique SPR tuning mechanism makes it possible to maintain the optical properties of PHAuNPs even after the loading of iron oxide.

As shown in Figure 4a, the particles can be dragged toward a permanent magnet, unequivocally indicating the magnetic characteristics of the Au nanoparticles. Hysteresis loop of dried particle powder is shown in Figure 4c. Since the Fe_3_O_4_ nanoparticles synthesized using the above-mentioned method are normally smaller than 20 nm, we expect to see a typical superparamagnetic behavior: zero remanence, zero coercivity, and a large saturation field. The small hysteresis shown in the measurement may reflect the presence of some large Fe_3_O_4_ nanoparticles (>30 nm) inside PHAuNPs. Given the size of the hollow space (>50 nm) and the thickness of the porous shell (25 nm), the inward diffusion of OH^-^ ions may be partially obstructed, resulting in a much slower nucleation rate. As such, the inside particles could grow large. The measured high saturation field is in consistence with the superparamagnetic characteristic. This suggests a mixture of superparamagnetic and ferromagnetic nanoparticles. Ferromagnetic nanoparticles are usually undesirable for bioapplications because of their agglomeration caused by magnetic attraction. However, for iron oxide nanoparticles-loaded PHAuNPs, the thick Au shell can effectively separate them far apart to avoid such magnetic aggregation.

## Conclusion

We have shown that the core/shell-structured Fe_3_O_4_/Au nanoparticles can be synthesized by trapping Fe_3_O_4_ nanoparticles inside hollow Au nanoparticles. Because the resulted composite nanoparticles combine the desirable magnetic and plasmonic properties into one nanoentity, they are particularly suitable for in vivo diagnostic and therapeutic applications, where the Au surface provides anchorage sites for attachment of functional molecules and the particles can be activated by both NIR light and magnetic field. As more and more hollow nanoparticles become available, we believe that this synthetic method would find general applications in the fabrication of core–shell multifunctional nanostructures.
